# A meta-analysis of diffusion tensor imaging of substantia nigra in patients with Parkinson’s disease

**DOI:** 10.1038/s41598-018-20076-y

**Published:** 2018-02-13

**Authors:** Xiao-Yan Deng, Li Wang, Ting-Ting Yang, Rui Li, Gang Yu

**Affiliations:** 1 Department of Neurology, The First Affiliated Hospital of Chongqing Medical University, Chongqing, China; 2 Department of Hemato-oncology, Chongqing University Cancer Hospital & Chongqing Cancer Institute & Chongqing Cancer Hospital, Chongqing, China; 3 Department of Neurology, The sixth people’s hospital of Chongqing, Chongqing, China

## Abstract

Parkinson’s disease (PD) is a common neurodegenerative disease characterized by severe, selective loss of pigmented neurons in the substantial nigra (SN). Previous studies have indicated that such loss could be detected by diffusion tensor imaging (DTI). Here, we try to consolidate current DTI data to both quantitatively determine the imaging changes in SN, as well as explore the potential use of DTI for PD diagnosis. Fourteen research articles are included in this meta-analysis, each obtained by searching PubMed, EMBASE, or Cochrane library database dated until July 2017. The articles contain 14 trials with 298 total PD patients and 283 healthy controls (HCs). The results show not only significantly lower FA values of SN in PD compared to that of HCs (WMD = −0.02, 95% CI = [−0.03, −0.02], p < 0.00001), but also a significantly higher MD in PD compared to HCs (WMD = 0.05, 95% CI = [0.03, 0.07], P < 0.0001). This indicates that the sharp difference detected between PD patients and HCs can be detected by DTI. By further analyzing the heterogeneity, we found that FA measurement of SN could be potentially used as a surrogate, noninvasive diagnostic marker toward PD diagnosis.

## Introduction

Parkinson’s disease (PD) is an age-related neurodegenerative disease with unknown etiology and ranks second behind Alzheimer’s diseases in prevalence. It mostly occurs in the elderly, with the prevalence of 1% in patients over 60 years old and 4% of those over 80 years old, and leads to severe social and economic burdens for both patients and caregivers^1,2^. Molecular and neuropathology findings have been fully documented for decades since the characterization of severe selective loss of pigmentation of neurons in SN associated with formation of Lewy bodies and neuritis in specific regions of the brain^3^. The clinical diagnosis of PD usually relies on hallmark symptoms, including bradykinesia, rigidity, resting tremors, and postural instability. Still, the early detection of PD is hindered by the fact that hallmark-based screening is only 53% accurate for those with <5 years of disease duration^4^. Thus, early diagnosis of PD was critical to initiate precise treatment by developing either bio- or image-markers that can be used as early diagnostic tools or to monitor the effectiveness of treatments. Until now, no such validated markers are available for clinical use, even though extensive investigations of this method have been carried out.

Diffusion Tensor Imaging (DTI) is a non-invasive, MRI-based neuroimaging technique that can analyze the diffusivity in brain tissues. The method is sensitive to the flow of water molecules as it predominately diffuses along axons. Fractional anisotropy (FA) is a measure that reflects the degree of diffusivity in different directions and can inform our understanding of the microstructural organization of the tensors. Mean diffusivity (MD) represents the overall movement of water molecules within the brain. Most often, DTI has been utilized to detect alterations in white matter, but it can also be used to detect grey matter abnormalities. It could be able to detect those changes earlier than the conventional structural MRI technique. Several DTI studies have reported alterations in diffusivity in subcortical structures and reduced FA in the SN with PD^5,6^. One study demonstrated DTI changes in mice treated with 1-methyl-4-phenyl-1,2,3,6-tetrahydropyridine (MPTP) that were correlated with the loss of SN dopamine neurons^7^. Studies have also shown that a decreased FA value may arise from demyelination, axonal loss, or changes in the size of axons^8^. The studies also show that structurally intact white matter has high FA but low MD, whereas structurally damaged white matter has reverse findings, i.e. low FA but high MD. Thus, DTI is commonly used in clinical practice to determine decreased FA and increased MD values, which are indicative of possible neurodegenerative diseases^9^. The aim of this study was to integrate published data to estimate the diagnostic threshold of values by DTI using standard meta-analysis methods.

## Results

### Literature search

**Figure 1:**
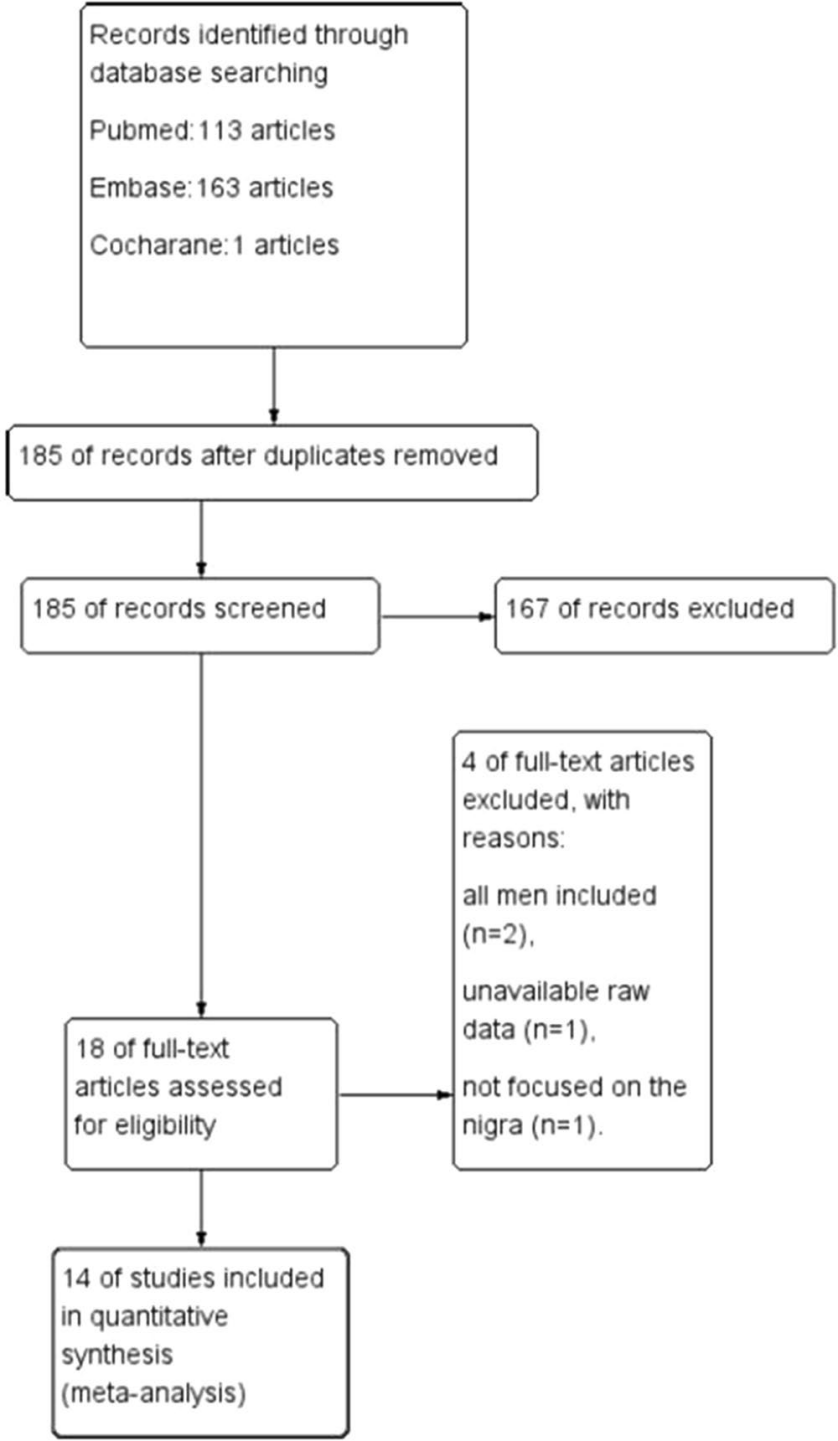
Flow diagram of study selection procedure.

By searching PubMed, EMBASE, and Cochrane library database dated until July 2017, a total of 277 relevant studies were retrieved. Of these, 92 were excluded as duplications and 167 were excluded after having found the reviewed titles, abstracts, and full texts off-topic. By carefully reviewing the remaining 18 studies, 4 were removed due to data from only male gender (2), unavailability of raw data (1), ordata not focused on nigra (1). Finally, 14 studies containing 298 PD patients and 183 HCs were used for subsequent pooled analysis^9–22^ (Fig. 1).

### Study Characteristics

Table 1 lists detailed information from 14 included studies published between 2007 and 2016 with sample sizes ranging between 20 and 151. Four^11,13,17,19^ of the studies were conducted in North America (3 in the USA, one in Canada), 6^9,12,15–17,21,22^ in Europe (3 in the UK, 2 in Italy, 1 in Sweden), 3^10,14,18^ in Asia (1 in China, 2 in Singapore), and one^20^ in Australia. Eight^9–13,15,16,20^ of the MRI scanners used in the studies were from Siemens, the rest^14,17–19,21,22^ from Philips, Allegra, and General Electrics. The magnetic field strength index of the MRI scanners used in nine^9,11,13–18,22^ of the studies was 3.0 Tesla, while the remaining five^10,12,19–21^ studies used 1.5 Tesla. Changes of both FA and MD were recorded in 12 studies, changes of FA only^11^ or MD only^12^ was recorded in one study each. According to the NOS (Newcastle-Ottawa assessment scale) quality assessment criteria, nine studies were defined as “good quality” (4 studies scored 8 and 5 studies scored 7), whereas five studies were listed as “fair quality” (scored 6). Detailed information of quality assessment was described in Table 2.

**Table 1:** Characteristics of included studies.

Author year	No.of PD/ controls	Country	Scanner	Field-str	Head coil	DTI dir	ROI placement	statistic significance	M-corr.	Eddy cur.
Li^14^ 2015	23/23	China	Philips	3.0 T	8	31	manual	FA	√	√
Perea^17^ 2013	12/13	USA	Allegra	3.0 T	?	36	manual	none	√	√
Schwarz^22^ 2013	32/27	UK	Philips	3.0 T	8	32	manual	MD	√	√
Scherfler^20^ 2013	16/14	Austrilia	Siemens	1.5 T	8	12	automatical	FA, MD	?	?
Du^11^ 2011	16/16	USA	Siemens	3.0 T	8	42	Semi–Manual	FA	√	√
Prakash^18^ 2012	11/12	Singapore	Philips	3.0 T	?	16	manual	FA, MD	?	?
Skorpil^21^ 2012	13/13	Sweden	Philips	1.5 T	16	32	manual	FA	√	√
Rolheiser^19^ 2011	14/14	Canada	General Electrics	1.5 T	8	31	manual	FA	√	√
Menke^15^ 2010	10/10	UK	Siemens	3.0 T	12	60	Semi–Manual	none	√	√
Péran^16^ 2010	30/22	Italy	Siemens	3.0 T	?	30	manual	FA	√	√
Gattellaro^12^ 2009	10/10	Italy	Siemens	1.5 T	4	12	manual	MD	?	?
Chan^10^ 2007	73/78	singapore	Siemens	1.5 T	?	12	manual	FA	√	√
Langley^13^ 2016	20/17	USA	Siemens	3.0 T	12	64	manual	none	?	√
Loane^9^ 2016	18/14	UK	Siemens	3.0 T	32	64	manual	none	√	√

Year: the year of the article published; No. of PD/controls: the number of PD patients compare to controls in article; Country: the country of the study carried out; Field-str: Field-strength of magnet; Head coil: number of channels within the receiver headcoil; DTI dir: Number of DTI diffusion directions for data acquisition; ROI placement: the method of ROI placement; statistic significance: If the FA or MD have statistic difference; M-corr: Motion correction used; Eddy cur: Eddy current correction used; “√” means a positive indicator; “?” means unclear.

**Table 2:** Assessment of study quality.

Reference	Quality Indications of Newcastle-Ottawa Scale								Total
	A	B	C	D	E	F	G	H	
Li 2015^14^	1	0	0	1	1	1	1	1	6
Perea 2013^17^	1	0	1	1	1	1	1	1	7
Schwarz 2013^22^	1	1	1	1	1	1	1	1	8
Scherfler 2013^20^	1	0	1	1	2	1	1	1	8
Du 2011^11^	1	0	0	1	1	1	1	1	6
Prakash 2012^18^	1	1	0	1	1	1	1	1	7
Skorpil 2012^21^	1	0	1	1	1	1	1	0	6
Rolheiser 2011^19^	1	0	1	1	1	1	1	1	7
Menke 2010^15^	1	0	0	1	1	1	1	1	6
Péran 2010^16^	1	0	1	1	1	1	1	1	7
Gattellaro 2009^12^	1	1	0	1	1	1	1	0	6
Chan 2007^10^	1	1	1	1	1	1	1	1	8
Langley 2016^13^	1	1	1	1	1	1	1	1	8
Loane 2016^9^	1	0	1	1	1	1	1	1	7

A. Adequate definition of Case; B. Representativeness of cases; C. Selection of control; D. Definition of control; E. Control for important factor or additional factor; F. Exposure assessment; G. Same method of ascertainment for cases and controls; H. Nonresponse rate.

### Primary Outcomes

#### Nigral FA changes

**Figure 2:**
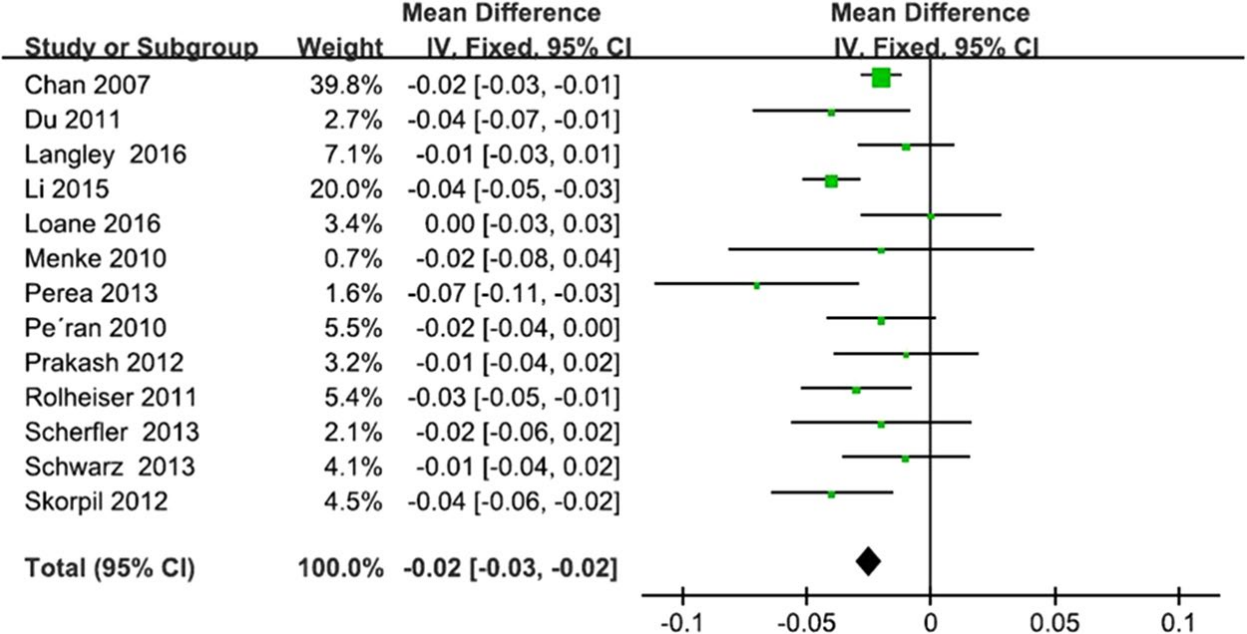
Forrest plot of PD induced nigral FA changes. IV, inverse variance; CI, confidence interval; Fixed, fixed-effect model.

Thirteen studies (288 PD patients and 273 HCs) reported nigral FA changes induced by PD that showed a significant FA reduction in SN of PD patients with an estimated WMD of −0.02 (95% CI = [−0.03, −0.02], p < 0.00001) (Fig. 2). No significant heterogeneity was recorded (I^2^ = 48%). The fixed-effect model was selected to calculate the pooled mean effect size.

#### Nigral MD changes

**Figure 3:**
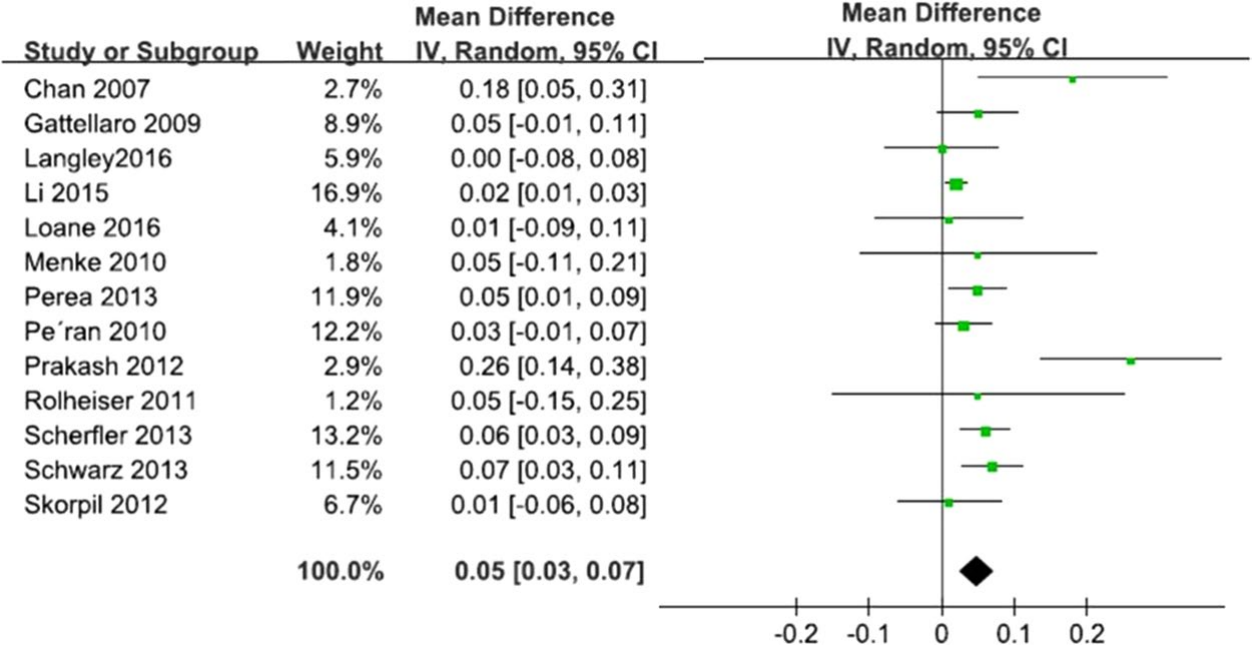
Forrest plot of PD induced nigral MD changes. Random, random-effect model.

Thirteen studies (282 PD patients and 267 HCs) reported nigral MD changes that showed a significant MD increase in PD patients compared to HCs, with an estimated WMD of 0.05 (95% CI = [0.03, 0.07], p < 0.0001) (Fig. 3). A moderate-level of heterogeneity was recorded (I^2^ = 58%). The random-effect model was used to calculate the pooled mean effect size.

#### Subgroup analysis of FA and MD changes

**Figure 4:**
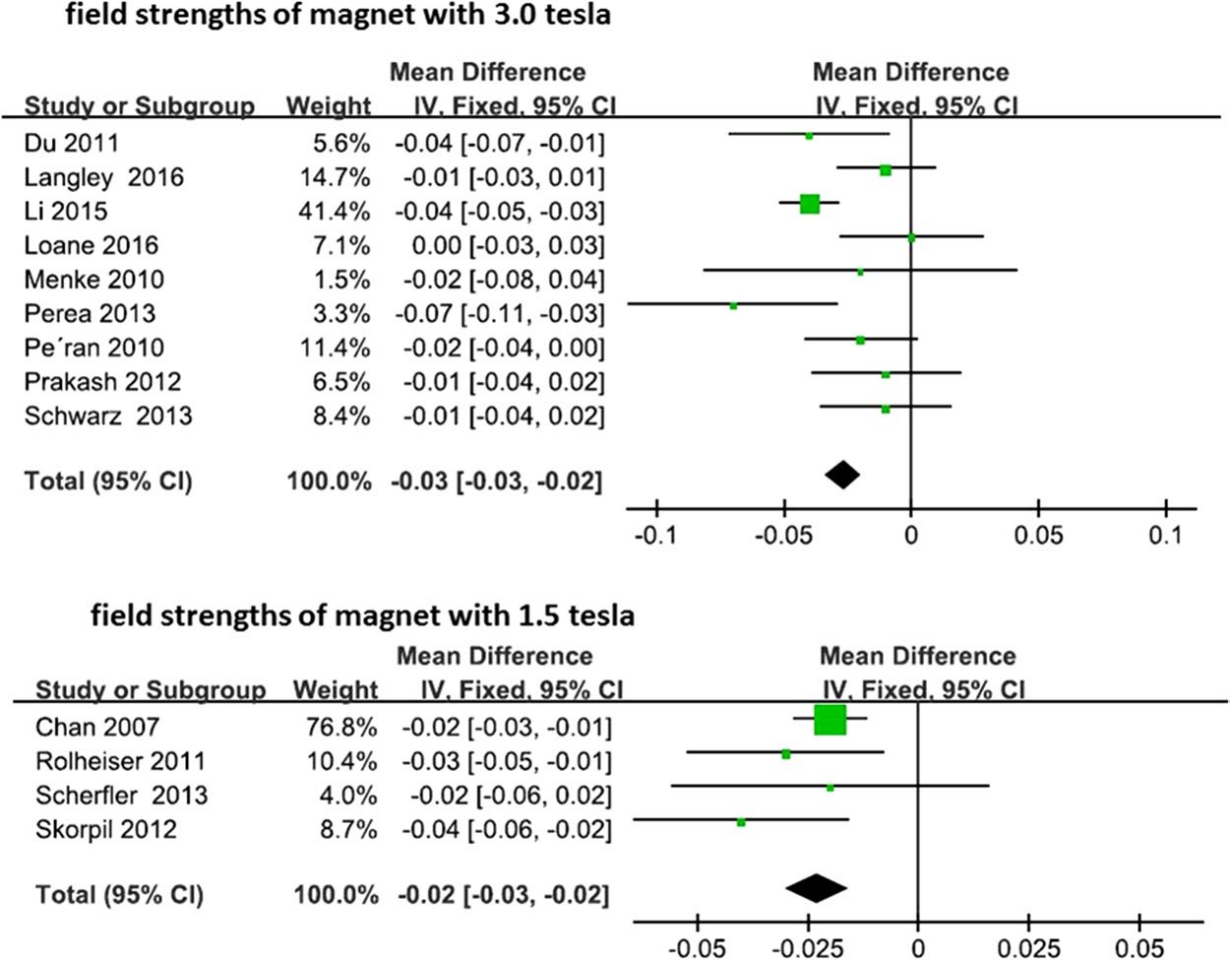
Forrest plot of subgroup analysis of PD induced nigral FA changes.

**Figure 5:**
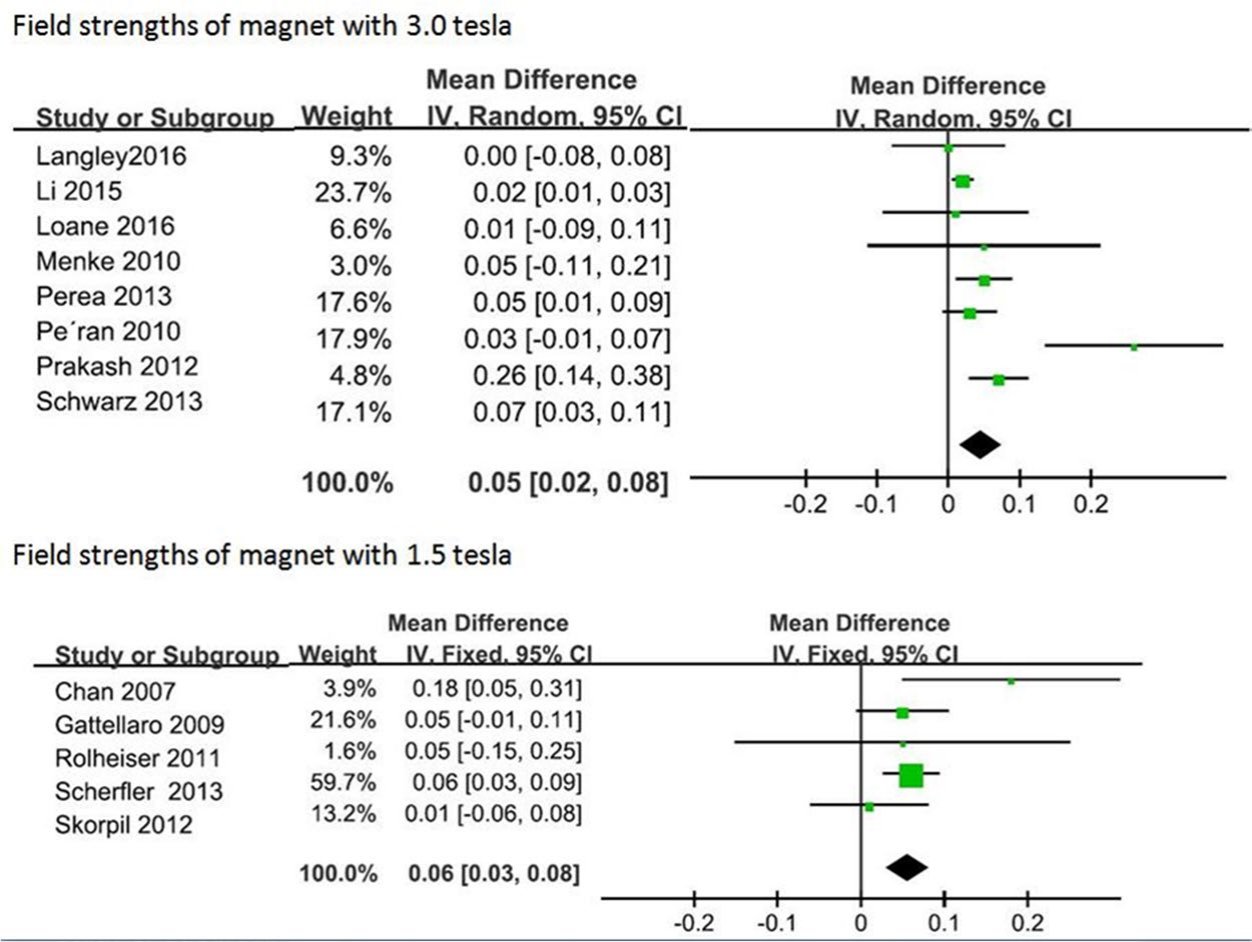
Forrest plot of subgroup analysis of PD induced nigral MD changes.

In terms of improved signal to noise ratio and increased image resolution, higher field strength magnets were generally considered to be more desirable and may have had an influence on imaging results. We therefore conducted a subgroup analysis based on differing field strengths. Among the thirteen studies with reported FA changes, nine studies used 3.0 Tesla. Analysis of the studies that utilized 3.0 Tesla field strength indicated a significant difference on FA changes between PD patients and HCs, with an estimated WMD −0.03 (95% CI = [−0.03, −0.02], p < 0.00001). Moreover the four studies that used a 1.5 Tesla field strength also documented a significant difference on FA changes in the meta-analysis, with an estimated WMD −0.02 (95% CI = [−0.03, −0.02], p < 0.0001) (Fig. 4). Among the thirteen studies that have reported MD changes, the meta-analysis of eight studies using 3.0 tesla field strengths and five studies using 1.5 tesla field strengths also indicated significance, with an estimated WMD of 0.05 (95% CI = [0.02, 0.08], p = 0.003) and 0.06 (95% CI = [0.03, 0.08], p < 0.0001), respectively (Fig. 5).

### Sensitivity analyses

Sensitivity analyses were performed by removing each study in turn and re-analyzed. No changes of significant differences were documented on DTI measurements (FA, MD) between the PD patients and HCs.

### Publication bias

**Figure 6:**
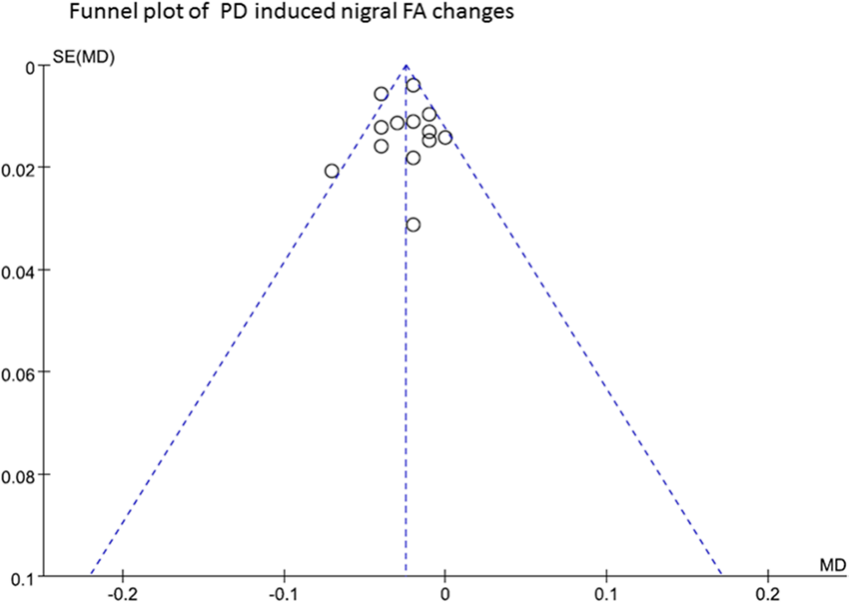
Funnel plot of PD induced nigral FA changes. SE, standard error; MD, mean differences.

**Figure 7:**
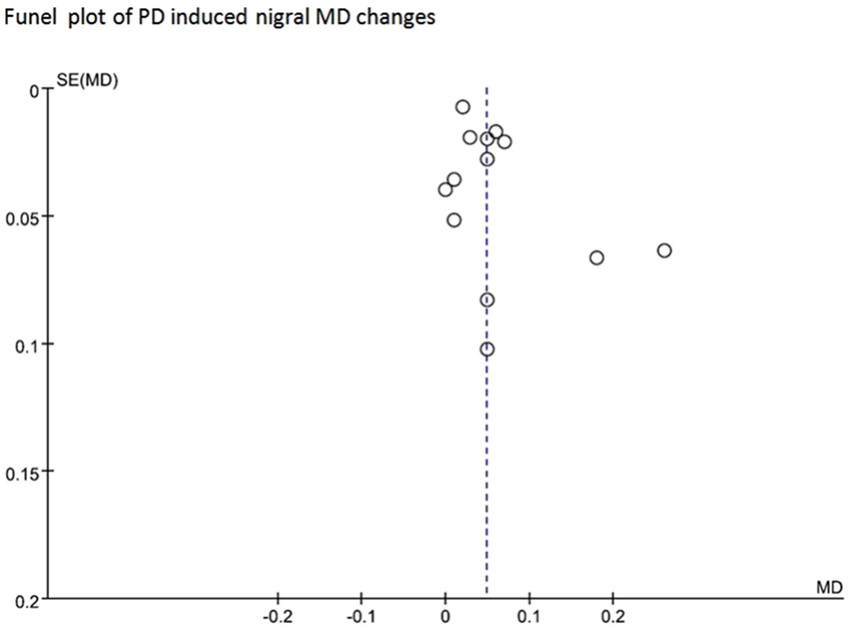
Funnel plot of PD induced nigral MD changes.

No significant publication bias was identified by Funnel Plot and Egger regression intercept test. The Funnel plots were symmetrical, and the Egger regression intercept test had no significant publication bias for the meta-analysis of FA changes (t = −1.41; p = 0.19) and MD changes (t = −0.46; p = 0.656). Detailed information was described in Figs 6, 7.

## Discussion

Current meta-analysis did find significantly lower FA values and higher MD values in PD patients over HCs. By means of analyses of heterogeneity, sensitivity, and publication bias, an obvious variation in results was found in the meta-analysis for PD-induced nigral MD changes, so performed a measurement of FA in SN that could be a potential surrogate, non-invasive marker for precise diagnosis of PD. To date, no DTI technique has been used to detect preclinical or early PD diagnosis, but one study on rodent model of PD showed reduced FA in the SN at its early stage that was correlated with the loss of SN dopamine neurons^7^. This result could provide us with clues to further investigate the screening of preclinical PD via DTI measurements.

There is a possibility that imaging heterogeneity in this meta-analysis could have arisen from differences in methodology, data acquisition, field strength of magnet, etc. across the 14 chosen studies. Indeed, such heterogeneity has also been found in this current meta-analysis concerning PD diagnosis, likely attributable to the different stages or severity of disease progression in patients imaged. These factors can unavoidably affect the homogeneity of imaging results. A recent study of nigral FA changes in PD patients couldn’t find any correlation between PD severity and FA changes^23^. Another possible source of heterogeneity in the data include the conversion of median averages and accompanying graphs into mean or variance via conversion tools. While we did not find publication bias in this meta-analysis, we cannot rule out that some biases went unreported in the sourced papers.

Regarding analysis by MRI imaging, the delineation of the SN was not uniform in included studies. For example, some studies focused their analysis on a sub-region of SN, while others used the whole SN as Region of Interest (ROI)^15,18^. If SN screening was based on ROI, the averaged value from SN couldn’t be compared among different studies. Prakash *et al*.^18^ described that lower FA and higher ADC were found at the left rostral SN compared to the right side. The subjective delineation of SN on T2-weighted MRI images resulted in most of the variation in the studies^22^. The primary pathology of PD is the loss of neuromelanin-generating dopaminergic neurons^24^ that can be manifested by MRI as hypodensity^25^, used by Langley *et al*.^13^ to depict the SN for screening. However, hypodensity can also be observed by iron deposition on SN in T2-weighted images. Defining the ROI of such scans could be difficult for DTI data analysis^25^. The SN volume delineated by neuromelanin sensitive MRI and T2w images differs not only in signal characteristics but also spatially^13^. The statuses of patients either during medication or withdrawal can influence the results. There are several examples of either the effect of withdrawal of PD medications for appraisal of disease severity by scanned MRI^14,23^, or on-medication state^12^, showed totally different MRI imaging results.

Two previous meta-analysis on DTI were published in 2013. The first analysis^24^ focused on the diagnostic value of DTI, with four of nine studies focusing on SN and other regions focusing on the globuspallidus, anterior olfactory structures, and cerebellar peduncles. The second analysis^22^ included 12 studies, eight of which have been included in this current meta-analysis, four having been excluded due to confounding components of each. Of the four excluded, three screened multiple sub-regions of SN^23,26,27^, making data collection difficult, and one focused on diffusion krutosis imaging (DKI) rather than DTI^28^. Our current study used careful review and evaluation to collect additional qualified studies that focus their analysis on the SN.

The following limitations of our study should be acknowledged: i) the limitation from the original data, we couldn’t further analyze with other impact factors such as different ages, genders, with or without target medications, and severity of disease itself. ii) The standard for study inclusion varied study by study, publication biases could still exist in our current study. iii) We couldn’t evaluate the sensitivity and specificity of the measure currently. Future high-quality and multicenter studies are warranted.

To date, we haven’t found any studies in the literature on using the DTI technique as a direct marker for PD diagnosis, preclinical or otherwise. Our meta-analysis showed that significantly lower FA values in SN could potentially be used as diagnostic marker for PD.

## Methods

### Literature search

Current meta-analysis was based on the guidelines of observational studies in epidemiology (MOOSE)^29^. PubMed, EMBASE, and Cochrane library were the sources of database used in our literature search dated until July 2017. Search terms used were “Parkinson’s disease”, “PD”, “Diffusion Tensor Imaging”, “DTI”, “Substantial Nigra”, “SN”, followed by the combinations of keywords and synonyms without language restrictions. Related references from search-out studies were also listed and reviewed to avoid possible overlook by our study. All papers have been screened by two authors who would be consensus or if the disagreement happened, the third author was consulted to decide if the study should be included or not. All included studies have been assessed by the Newcastle-Ottawa Scale (NOS) that was recommended by the Cochrane collaboration to guarantee the quality of each study^30^.

### Inclusion/Exclusion criteria

Criteria for inclusion: 1) the study was a case-control or cohort study; 2) the study should use DTI technique to screen on substantianigra in both PD patients and healthy controls; 3) the study should have the detailed data on FA, and MD or ADC (MD and ADC were not entirely equal, but both indexes were the descriptors of total magnitude of diffusion and should all be included in the analysis); 4) for longitudinal trials, only the data from the first period was included. Review articles, comments, editorials, and case reports were all excluded in our study.

### Data extraction

The following information was used in our meta-analysis, documented case by case: i) the authors, publication date, the number of participants, ages, disease duration, Unified Parkinson’s Disease Rating Scale (UPDRS) motor scores, Hoehn-Yahr (H-Y) stage, and medications; ii) MRI scanner parameters, including acquisition characteristics, field strength of the MRI scanner, head coil, voxel size, and DTI directions; iii) the method of delineating the neuroanatomy of SN and the way of DTI processing the analyzing; iv) primary data value outcomes (FA, MD or ADC). If the original studies provided with only parameters like median, range or quartiles instead of mean and standard deviation, the conversion procedures were performed and estimated by previously published conversion techniques^31^ to conform with the meta-analysis used previously^22^.

### Statistical analysis

The pooled weighted mean difference (WMD) and their 95% confidence intervals (CI) were calculated by the method of inverse variance with a random-effect or fixed-effect model. Statistical heterogeneity was evaluated using Higgins’ I^2^-statistics, in which I^2^ > 50% was considered heterogeneous among included studies. If I^2^ < 50%, a fixed-effect model was applied further, otherwise, a random-effect model was used instead. Funnel plot symmetry and Egger’s tests were used to evaluate the publication bias. Review Manager 5.3 and stataSE12.0 software were used to perform data analyses in current study. P value < 0.05 was considered statistically significant.

### Disclosure

We would like to declare that the work has not been published previously. On behalf of all authors, the corresponding author states that there is no conflict of interest.

## Conclusion

This meta-analysis on measurements of FA in SN by DTI technique finds lower FA values in PD patients compared to the HCs. The strength of this finding indicates that FA measurement though DTI could be potentially used as a surrogate (complementary), non-invasive marker to diagnose PD more precisely. DTI could also be used to monitor the changes of both the brain structure and its functions for PD patients. Future research should focus on DTI measurement of image changes for early PD lesions, as well as its possible use as a preclinical diagnostic tool for those at risk of developing PD.
